# Investigating the Antiviral Properties of *Nyctanthes arbor-tristis* Linn against the Ebola, SARS-CoV-2, Nipah, and Chikungunya Viruses: A Computational Simulation Study

**DOI:** 10.3390/ph17050581

**Published:** 2024-04-30

**Authors:** Raed Albiheyri, Varish Ahmad, Mohammad Imran Khan, Faisal A. Alzahrani, Qazi Mohammad Sajid Jamal

**Affiliations:** 1 Department of Biological Sciences, Faculty of Science, King Abdulaziz University, Jeddah 21589, Saudi Arabia; 2 Centre of Excellence in Bionanoscience Research, King Abdulaziz University, Jeddah 21589, Saudi Arabia; 3 Health Information Technology Department, The Applied College, King Abdulaziz University, Jeddah 21589, Saudi Arabia; vaahmad@kau.edu.sa; 4 Centre for Artificial Intelligence in Precision Medicine, King Abdulaziz University, Jeddah 21589, Saudi Arabia; 5 Research Center, King Faisal Specialist Hospital and Research Center, Jeddah 21499, Saudi Arabia; mikhan@kfshrc.edu.sa; 6 Department of Biochemistry, Faculty of Science, Embryonic Stem Cell Unit, King Fahad Center for Medical Research, King Abdulaziz University, Jeddah 21589, Saudi Arabia; faahalzahrani@kau.edu.sa; 7 Department of Health Informatics, College of Applied Medical Sciences, Qassim University, Buraydah 51452, Saudi Arabia

**Keywords:** *Nyctanthes arbor-tristis*, phytochemicals, broad-spectrum antiviral, molecular docking simulation, molecular dynamics simulation

## Abstract

*Background:* The hunt for naturally occurring antiviral compounds to combat viral infection was expedited when COVID-19 and Ebola spread rapidly. Phytochemicals from *Nyctanthes arbor-tristis* Linn were evaluated as significant inhibitors of these viruses. *Methods:* Computational tools and techniques were used to assess the binding pattern of phytochemicals from *Nyctanthes arbor-tristis* Linn to Ebola virus VP35, SARS-CoV-2 protease, Nipah virus glycoprotein, and chikungunya virus. *Results:* Virtual screening and AutoDock analysis revealed that arborside-C, beta amyrin, and beta-sitosterol exhibited a substantial binding affinity for specific viral targets. The arborside-C and beta-sitosterol molecules were shown to have binding energies of −8.65 and −9.11 kcal/mol, respectively, when interacting with the major protease. Simultaneously, the medication remdesivir exhibited a control value of −6.18 kcal/mol. The measured affinity of phytochemicals for the other investigated targets was −7.52 for beta-amyrin against Ebola and −6.33 kcal/mol for nicotiflorin against Nipah virus targets. Additional molecular dynamics simulation (MDS) conducted on the molecules with significant antiviral potential, specifically the beta-amyrin-VP35 complex showing a stable RMSD pattern, yielded encouraging outcomes. *Conclusions:* Arborside-C, beta-sitosterol, beta-amyrin, and nicotiflorin could be established as excellent natural antiviral compounds derived from *Nyctanthes arbor-tristis* Linn. The virus-suppressing phytochemicals in this plant make it a compelling target for both in vitro and in vivo research in the future.

## 1. Introduction

*Nyctanthes arbor-tristis* (*N. arbortristis*) is a plant belonging to the *Oleaceae* family and the *Nyctanthes* genus. It is traditionally used for its therapeutic properties in tropical and subtropical regions of South and Southeast Asia. It grows in the outer Himalayas of India. It can be found in areas such as Jammu and Kashmir, Assam’s east coast, Bengal, and Tripura, which stretch across the country’s central region and down to the Godavari River in the south, as well as in Nepal. It is typically a tiny shrub or tree with beautiful, incredibly fragrant flowers that bloom at night and fade before the sun rises, its red and white colors creating a lovely contrast with the ground below. The plant is called the “Tree of Sadness” (*arbor-tristis*) since it completely loses its brightness during the day. Other names for it include Night-flowering Jasmine, Coral Jasmine, Parijat, Queen of the Night, and Harsinghar [1]. Typically, flowering takes place from July to October. *N. arbor-tristis* favors a remote location with some shade for growth. The crude extracts of the plant’s leaves, stems, and flowers are reported to contain key classes of photochemicals, such as glycosides, flavonoids, oleanolic acid, tannic acid, aromatic oils, lupeol, friedeline, carotene, benzoic acid, and glucose, with significant hepatoprotective, hair tonic, antiviral, anti-leishmaniasis, anti-histaminic, antipyretic, antimalarial, antimicrobial, and anti-inflammatory effects [2].

Eddie’s Hot Plate and Tail Clip analysis revealed that *Nyctanthes arbor-tristis* leaf extracts in hydro ethanol, ethanol, chloroform, and aqueous solutions had considerable narcotic analgesic efficacy. The hydroethanolic extract in the investigation at 1000 mg/kg body weight showed a superior analgesic effect to that of the other three extracts [3]. A computational study also showed that the phytochemicals significantly interacted with the Janus kinase (JAK) enzymes involved in rheumatoid arthritis [4]. Viral illnesses like polio, mumps, acquired immunodeficiency syndrome (AIDS), severe acute respiratory syndrome (SARS), encephalitis, measles, Middle East respiratory syndrome (MERS), dengue fever, chikungunya fever, influenza, and coronavirus disease 2019 (COVID-19) have historically caused significant suffering in humans. Some viral strains, like Epstein–Barr, hepatitis B, hepatitis C, lymphotropic virus type 1 of human T cells, papillomaviruses, and Kaposi’s sarcoma-associated herpesvirus, can cause critical infection and are among those causing cancer in humans [1,5]. The computational studies explored in the recently described review stated that phytonutrients, namely, silibinin (a flavonolignan), lycorine (a pyrrolidine alkaloid), suramin, theaflavin (a tea polyphenol), corilagin (a gallotannin), baicalein (5,6,7-trihydroxyflavone), and hesperidin (a citrus bioflavonoid) exhibit a broader spectrum of antiviral activity compared to favipiravir or remdesivir [6].

SARS-CoV-2 is anticipated to keep spreading and pose a severe threat to our civilization, much like the virus responsible for the common cold. Antiviral drugs are necessary to safeguard those at the highest risk, heal infected patients, and administer prophylactically. Notwithstanding the complicated pharmaceutical discovery process, finding therapeutic drugs that inhibit coronavirus replication must be emphasized since they can improve the lives of millions worldwide.

Furthermore, the development of vaccines has evolved substantially, but supply and timing are currently preventing their widespread use. Numerous vaccines have been created and approved for widespread immunization [7]. However, some vaccinations may be too expensive for developing countries to store at cryogenic temperatures. The effectiveness of vaccinations to combat the virus may also be impacted by several modifications to the viral genome [8,9].

Recent cases of COVID-19 have shifted attention toward traditional plant-based medicines, which can be found in many regions across the globe. To manage COVID-19, various drug targets, like spike and envelope proteins, membrane proteins, nucleocapsid proteins, endosomal pH, angiotensin-converting-enzyme (ACE) inhibitors, and proteases, have been explored. The hunt for naturally occurring antiviral compounds to combat viral infection was expedited when COVID-19 spread rapidly [7]. Many in vitro, in vivo, and in silico investigations were carried out on plants and repurposed pharmaceuticals to find possible therapeutic compounds against the virus.

Even though in vitro studies help comprehend the biology of viruses in highly controlled settings, these models typically fail to reproduce the complexity of natural biological systems faithfully. However, animal experiment-based research is expensive; it necessitates biosafety level 3 (BSL-3) animal facilities and creates ethical issues. It is intriguing to target proteases such as PLpro and 3CLpro, VP35 of the Ebola virus, the glycoprotein of the Nipah virus, and the nsP2 protease of chikungunya to develop effective broad-spectrum antiviral medications. These targets make essential contributions to the development of viral disease. The Ebola virus is a highly deadly infectious agent capable of infecting people. The Ebola virus protein VP35 hinders the synthesis of host interferon (IFN)-α/β by disrupting the host’s immunological response to viral invasion. As a result, it is seen as a potential therapeutic target. The viral protein VP35 inhibits the activity of human-type I IFNs to suppress the response of dendritic cells (DCs) to the viral infection. This mechanism helps the virus avoid the buildup of signal transducers, specifically activators of transcription 1 (STAT1), in the infected cells. Moreover, VP35 inhibits dsRNA-dependent protein kinase receptor (PKR) activation, which is responsible for the production of IFN [10].

The glycoprotein NiV-G attached to the exterior of the infectious agent is crucial for identifying the ephrin-B2 (EFNB2) and ephrin-B3 (EFNB3) sensors on the surface of host cells. This interaction facilitates the binding of the pathogen to the cells, resulting in the fusion of their membranes and viral entry into the host. Therefore, the molecular suppression of NiV-G is a key approach for the control of NiV infection [11]. The nsP2 cysteine protease has been identified as a vital target for drugs in the fight against illnesses associated with Alphaviruses, such as the chikungunya virus, since it plays a key role in viral replication.

Thus, in the search for antiviral molecules derived from *Nyctanthes arbor-tristis*, phytochemicals were analyzed against targets from different viruses through computational screening, interaction analysis, and molecular dynamics simulation (MDS) studies.

## 2. Results

We used computational techniques like molecular docking, ADMET analysis, and MDS for the study. The results are discussed in the following sections.

### 2.1. Docking Data Interpretation

After performing the AutoDock tool’s docking analysis, we selected the top two natural compounds based on binding energies for further ADMET and interaction modeling analyses. The results showed that the natural compounds had interaction energies ranging between −9.11 and −3.73 kcal/mol compared with remdesivir (−6.8 kcal/mol) (see Table 1 and Supporting Information (Table S1)).

The binding energy values for selected natural compounds arborside-C and beta-sitosterol with the main protease were found to be −8.85 and −9.11 kcal/mol, respectively. At the same time, the control drug, remdesivir, had a value of −6.18 kcal/mol.

**Figure 1:**
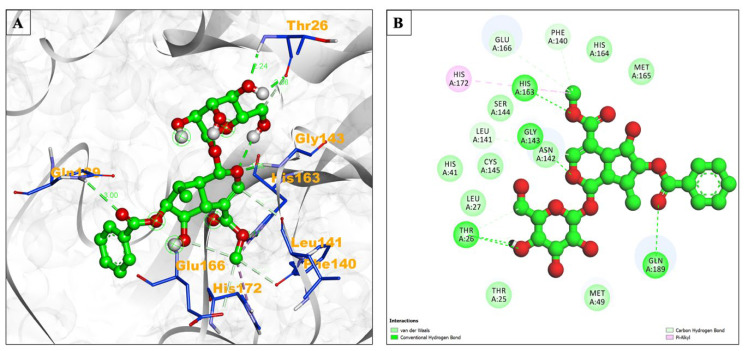
(**A**) Interacting site of arborside-C (central green ball and stick pattern) with the SARS-CoV-2 main protease (surrounding grey flat ribbon pattern). The blue stick pattern shows the interacting amino acid residues of the binding pocket, and green dotted lines show hydrogen bond formation. (**B**) The 2D graphic of amino acid residues involved in the interactions, shown by spheres.

Hydrogen bond formation facilitates molecular receptor–ligand interaction and affects several biological processes, including protein folding and catalysis [12,13]. Therefore, we also analyzed the hydrogen bonds between selected molecules. The main protease, arborside-C, formed a total of nine hydrogen bonds compared to the control drug remdesivir–protease interaction, which included five hydrogen bonds. In contrast, beta-sitosterol formed only one hydrogen bond. Furthermore, observed inhibition constants generated by AutoDock tool calculation were 456.39 nM, 210.29 nM, and 166.35 μM, respectively, for arborside-C, beta-sitosterol, and remdesivir (Table 1). Arborside-C formed a Van der Waals interaction with Leu27, Thr25, His41, Ser144, Met49, Cys145, Met165, and His164. His172 was also included in the Pi-Alkyl bond formation (Figure 1A,B).

**Figure 2:**
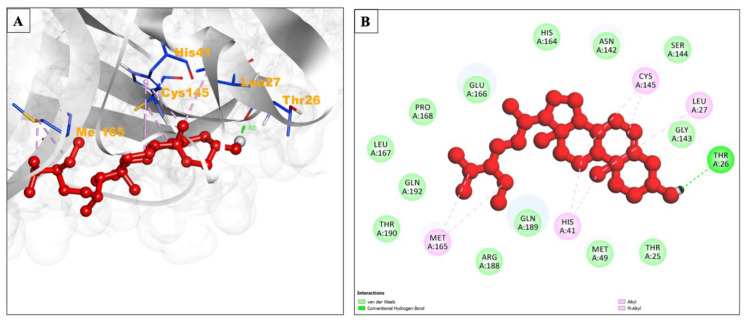
(**A**) Interacting site of the binding site of beta-sitosterol (central ball and stick pattern) with the SARS-CoV-2 main protease (surrounding grey flat ribbon pattern). The blue stick pattern shows the interacting amino acid residues of the binding pocket, and green dotted lines show hydrogen bond formation. (**B**) The 2D graphic of amino acid residues involved in the interactions, shown by spheres.

Beta-sitosterol formed Alkyl/Pi-Alkyl with amino acid resides Leu27, His41, Cys145, and Met165, while Thr25, Met49, Gly143, Asn142, Ser144, Glu166, Pro168, His164, Leu167, Arg188, Thr190, Gln192, and Gln189 produced Van der Waals interaction (Figure 2A,B).

**Figure 3:**
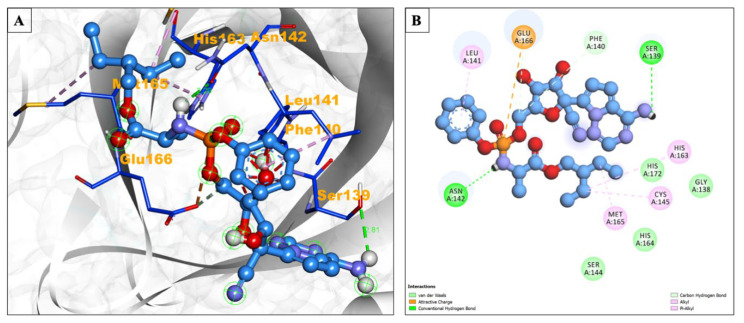
(**A**) The interacting site of remdesivir (central light blue ball and stick pattern) with the SARS-CoV-2 main protease (surrounding grey flat ribbon pattern). The blue stick pattern shows the interacting amino acid residues of the binding pocket, and green dotted lines show hydrogen bond formation. (**B**) The 2D graphic of amino interactions, shown by spheres.

During remdesivir’s interaction with the main protease, amino acid residues Gly138, Ser144, His164, and His172 interacted via Van der Waals forces, while Leu141, Cys145, His163, and Met165 formed Alkyl/Pi-Alkyl bonds; Glu166 also showed an attractive charge (Figure 3A,B).

**Figure 4:**
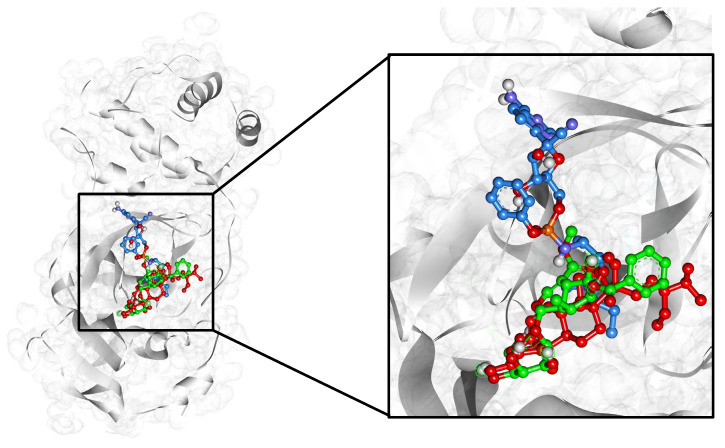
3D visualization of the SARS-CoV-2 main protease (solid grey surface) binding pocket containing arborside-C (green), beta-sitosterol (red), and the control drug remdesivir (blue), shown as ball and stick patterns in the center.

Selected compounds, including remdesivir, were found to interact at the same active site with slight deviations (Figure 4).

Virtual screening revealed that beta-amyrin interacted well with Ebola virus VP35 (bound to a small molecule with PDB ID 4IBB). The comparative results with remdesivir are summarized in the following table.

Remdesivir exhibited a binding energy of −3.98 kcal/mol. It formed three hydrogen bonds. The inhibition constant for remdesivir was 1.21 mM. Amino acid residues Val221, Val245, Gln244, Val294, Pro293, Asp302, and Phe328 were involved in hydrophobic interactions. A Pi-Sigma interaction with Lys248 and Alkyl/Pi-Alkyl interaction with Ile295 and Ile297 were formed (Table 2).

**Figure 5:**
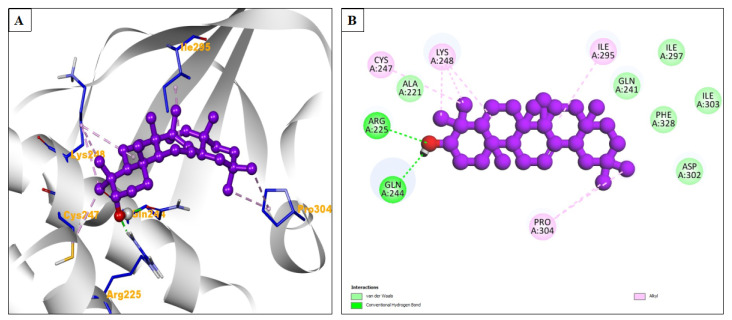
(**A**) The interacting site of beta-amyrin (central purple ball and stick pattern) with Ebola virus VP35 bound to a small molecule (PDB ID: 4IBB, surrounding grey flat ribbon pattern). The blue stick pattern shows the interacting amino acid residues of the binding pocket, and green dotted lines show hydrogen bond formation. (**B**) The 2D graphic of amino interactions, shown by spheres.

Beta-amyrin demonstrated a more favorable binding energy of −7.52 kcal/mol compared to remdesivir. It formed two hydrogen bonds. Beta-amyrin had a lower inhibition constant of 3.08 μM, suggesting potentially higher efficacy in inhibiting Ebola virus VP35. Amino acid residues Cys247, Lys248, Ile295, and Pro304 formed alkyl interactions, while amino acid residues Ala221, Gln241, Phe328, Asp302, Ile303, and Ile297 contributed to the binding via hydrophobic interactions (Figure 5A,B).

Further, Table 3 details the molecular interaction between the control drug remdesivir and the natural compound nicotiflorin, which interacts with the Nipah G attachment glycoprotein (PDB ID: 3D11).

**Figure 6:**
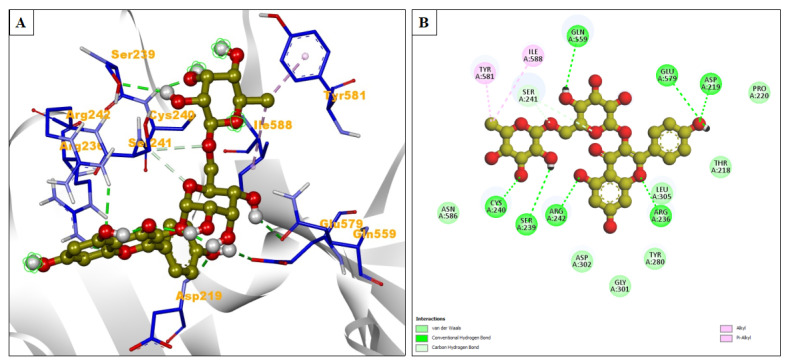
(**A**) The interacting site of nicotiflorin (central olive ball and stick pattern) with Nipah G attachment glycoprotein (PDB ID: 3D11, surrounding grey flat ribbon pattern). The blue stick pattern shows the interacting amino acid residues of the binding pocket, and green dotted lines show hydrogen bond formation. (**B**) The 2D graphic of amino interactions, shown by spheres.

Remdesivir showed a binding energy of −6.27 kcal/mol, indicating its effectiveness in binding with the protein. It formed five hydrogen bonds. The inhibition constant for remdesivir was 25.29 μM, suggesting moderate binding affinity. Hydrophobic interactions were formed by amino acid residues. Pro353, Gly352, Cys282, Tyr351, Phe458, Trp504, Leu305, Arg242, Asp219, Pro220, Val507, Thr218, Ser241, and Leu234 were involved in hydrophobic interactions. Other interactions also formed, like Pi-Alkyl with Tyr280 and Pro441, Pi-Sigma with His281, Pi-Cation with Lys560, Pi-Anion with Asp302, and attractive charge with Asp302 (Table 3). The natural compound nicotiflorin exhibited a slightly higher binding energy of −6.39 kcal/mol, which could imply a stronger binding affinity. It formed eight hydrogen bonds during the interaction. Nicotiflorin had a lower inhibition constant of 20.63 μM, further supporting the notion of it having a stronger binding affinity. Amino acid residues Asn586, Asp302, Gly301, Tyr280, Leu305, Thr218, and Pro220 were involved in hydrophobic interactions. Alkyl/Pi-Alkyl contact was formed by Ile588 and Tyr581 (Figure 6A,B).

While both compounds exhibited significant interactions with the Nipah G attachment glycoprotein, nicotiflorin may offer enhanced binding characteristics, as evidenced by its higher binding energy and lower inhibition constant. Further investigation into nicotiflorin’s antiviral potential is warranted.

The molecular interaction analysis summarized in Table 4 provides insights into the interactions between the natural compound beta-amyrin, the control drug remdesivir, and the chikungunya virus nsP2 protease (PDB ID: 3TRK).

**Figure 7:**
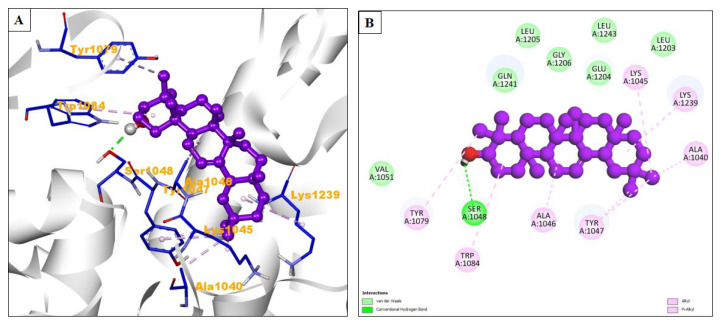
(**A**) The interacting site of beta-amyrin (central purple ball and stick pattern) with the chikungunya virus nsP2 protease (PDB ID: 3TRK, surrounding grey flat ribbon pattern). The blue stick pattern shows the interacting amino acid residues of the binding pocket, and green dotted lines show hydrogen bond formation. (**B**) The 2D graphic of amino acid interactions, shown by spheres.

Remdesivir exhibited a binding energy of −5.48 kcal/mol and formed three hydrogen bonds. Its inhibition constant was 95.82 μM, suggesting a moderate binding affinity. The amino acid residues His1236, Ala1237, Lys1045, Tyr1047, Tyr1079, Leu1205, Trp1084, Pro1049, Lys1091, Glu1050, Arg1271, Thr1268, Leu1243, and Glu1204 were involved in hydrophobic interactions. Lys1239, Ala1046, and Val1051 were involved in Alkyl/Pi-alkyl interactions (Table 4). The natural compound beta-amyrin exhibited a significantly lower binding energy of −8.54 kcal/mol, indicating a potentially stronger binding affinity. It formed one hydrogen bond with a length of 2.11674 Angstrom. Beta-amyrin had a lower inhibition constant of 551.06 nM, suggesting that it might be more effective in inhibiting the chikungunya virus nsP2 protease. Amino acid residues Val1051, Gln1241, Leu1205, Gly1206, Glu1204, Leu1243, and Leu1203 created hydrophobic interactions, while Tyr1079, Trp1084, Ala1046, Tyr1047, Ala1040, Lys1239, and Lys1045 were involved in Alkyl/Pi–Alkyl interactions (Figure 7A,B).

The data suggest that beta-amyrin could potentially be more effective than remdesivir in inhibiting the chikungunya virus nsP2 protease due to its lower Ki and higher binding energy. Both compounds exhibited hydrophobic interactions but differed in the residues involved, which could play a role in their respective efficacies and specificities. Further experimental validation is required to confirm these findings and explore potential therapeutic applications.

Overall, each compound interacted differently and efficiently with the viral proteins, and their binding energies and inhibition constants varied. All the selected compounds showed promising results, justifying further investigation for potential antiviral applications.

### 2.2. Drug-Likeness and ADMET Analysis

**Figure 8:**
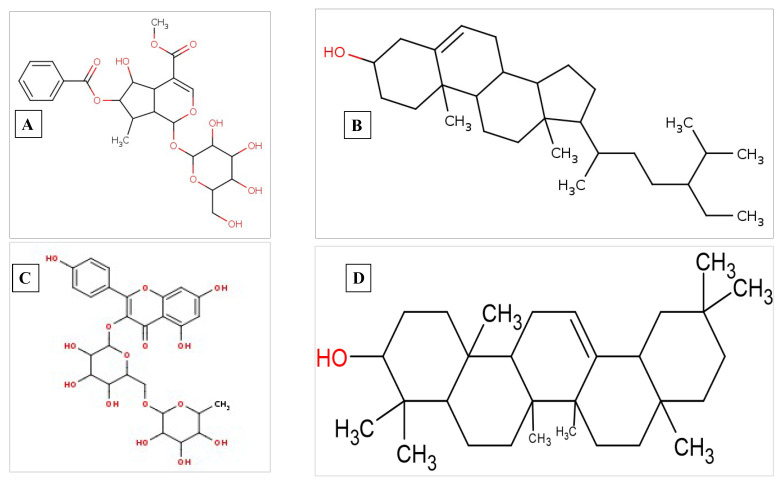
2D graphical representation of (**A**) arborside-C, (**B**) beta-sitosterol, (**C**) nicotiflorin, and (**D**) beta-amyrin. The 2D structures were generated by the Smi2Depict online tool [14].

After examining several parameters, including blood–brain barrier (BBB) permeability, gastrointestinal absorption, inhibition of cytochrome, substrate interaction with P glycoprotein, and log Kp value for skin permeation, arborside-C showed significant results and nearly identical data to those of the control drug remdesivir based on the ADME data that were obtained for beta-sitosterol, arborside-C, nicotiflorin, and beta-amyrin (Figure 8A–D) from the SwissADME server (see Supporting Information (Table S2)).

According to a drug-likeness analysis, the compound arborside-C showed two violations of Lipinski’s rule of five requirements, similar to the control drug [15]. Furthermore, the synthetic accessibility value and the bioavailability score fell within the predetermined cutoff range (see Supporting Information (Table S3)).

Regarding the Lipinski rule of five, arborside-C violated two criteria: it had a molecular weight greater than 500 g/mol and more than nine rotatable bonds. However, it did not infringe on any of the other criteria. Therefore, arborside-C was considered a “drug-like” molecule.

Arborside-C had a bioavailability score of 0.11, which indicated that it was poorly absorbed into the bloodstream. This is likely due to its high lipophilicity. Arborside-C also had a synthetic accessibility score of 6.14, which indicated that it was relatively easy to synthesize.

Beta-sitosterol did not violate any criteria of the Lipinski rule of five. Therefore, it was considered a “drug-like” molecule.

Beta-sitosterol had a bioavailability score of 0.55, indicating that it was well absorbed into the bloodstream. This is likely due to its low lipophilicity. Beta-sitosterol also had a synthetic accessibility score of 6.3, suggesting that it was relatively easy to synthesize.

Overall, remdesivir, arborside-C, and beta-sitosterol are all small-molecule drugs shown to have potential for treating COVID-19. Remdesivir and arborside-C are relatively easy to synthesize but have low bioavailability. Beta-sitosterol is more bioavailable but more challenging to synthesize. Further research is needed to determine which of these compounds is most effective and safe for the treatment of COVID-19. Additionally, the toxicity observation of this study was completed using the server pkCSM (http://biosig.unimelb.edu.au/pkcsm/theory, accessed on 1 March 2024). Arborside-C is a non-toxic compound. It met the requirements established by factors like AMES toxicity, liver toxicity, sensitization of skin, toxicity of *T. pyriformis*, and minnow toxicity (see Supporting Information (Table S4)).

Arborside-C is not mutagenic, as indicated by its negative AMES test result. It had an acceptable dose of −0.397 mg/kg/day in humans. Arborside-C is not a hERG I or hERG II inhibitor. It had an oral acute toxicity (LD50) of 2.701 mg/kg and oral chronic toxicity (LOAEL) of 4.723 mg/kg in rats. Arborside-C does not cause hepatotoxicity or skin sensitization. It had a *T. pyriformis* toxicity of 0.285 log µg/L and a minnow toxicity of 3.731 log LC50.

Beta-sitosterol has been shown to have several health benefits, including reducing cholesterol levels, improving heart health, and relieving the symptoms of benign prostatic hyperplasia (BPH). Beta-sitosterol is mutagenic above 0.559 mg/kg/day in humans, as reflected by its positive AMES test. Beta-sitosterol is not an hERG I or hERG II inhibitor. It has an LD50 of 2.482 mg/kg and a LOAEL of −0.515 mg/kg. Beta-sitosterol does not cause hepatotoxicity or skin sensitization. It has a *T. pyriformis* toxicity of 0.285 log µg/L and a minnow toxicity of 4.614 log LC50.

Overall, remdesivir and arborside-C are both relatively safe compounds. They are not mutagenic and do not cause hepatotoxicity or skin sensitization. However, they are both moderately toxic, with oral acute toxicity (LD50) values of 2.329 mg/kg and 2.701 mg/kg, respectively, in rats. Beta-sitosterol is less harmful than remdesivir or arborside-C, with an oral acute toxicity (LD50) value of 2.482 mg/kg in rats. Additional investigation is required to ascertain the extended-term safety of arborside-C and beta-sitosterol.

We also predicted the ADMET properties of beta-amyrin and nicotiflorin. Both beta-amyrin and nicotiflorin were predicted to have low gastrointestinal absorption. They were predicted to be non-permeable to the BBB. Neither was expected to be a substrate for P-glycoprotein, a transporter protein that can limit drug absorption. Neither compound was predicted to inhibit any of the major enzymes of the cytochrome P450 (CYP) system involved in drug metabolism. The prediction of BBB non-permeability suggests that these compounds may not pass the BBB to reach the central nervous system. The value was −2.41, indicating low skin permeation. Negative log Kp values correspond to less skin permeation of the molecule. The value was significantly lower at −9.91, indicating even less skin permeation than beta-amyrin. This could be advantageous for some therapeutic applications, as it would reduce the risk of side effects on the central nervous system.

Beta-amyrin and nicotiflorin showed a greater binding affinity for other viral receptors and were also screened for drug-likeness using the SwissADME server. Beta-amyrin had a molecular weight (MW) of 426.72 g/mol and a total polar surface area (TPSA) of 20.23 A^2^. Nicotiflorin had an MW of 594.52 g/mol and a TPSA of 249.20 A^2^. Both compounds violated the Muegge size principle. Beta-amyrin had zero rotatable bonds and satisfied all other filtering criteria from the SwissADME server. Nicotiflorin had six rotatable bonds and violated filters for the Ghose, Veber, and Egan rules. The bioavailability score was 0.55 for beta-amyrin and 0.17 for nicotiflorin.

Both compounds exhibited distinct drug-likeness profiles. Beta-amyrin leaned toward lipophilicity and moderate oral absorption potential, while nicotiflorin was more polar, water-soluble, and challenging to synthesize. These insights can guide researchers in selecting promising drug candidates. Beta-amyrin and nicotiflorin were observed to be safe, as predicted by the results from the pkCSM server. A comparison of both compounds revealed no AMES mutagenicity.

The maximum tolerated dose (human) of beta-amyrin was −0.312, and that of nicotiflorin was 0.221. Nicotiflorin had a slightly higher tolerated dose. Neither compound inhibits hERG I. Both compounds inhibit hERG II.

The oral acute toxicity (LD50) parameter value for beta-amyrin was 2.296 in rats, suggesting a relatively low acute toxicity, while that of nicotiflorin was 2.518. Both compounds had comparable acute toxicity. Beta-amyrin’s LOAEL value was 0.922, indicating chronic exposure tolerance, while nicotiflorin’s LOAEL value was significantly higher at 5.488, suggesting better tolerance in chronic exposure scenarios. Nicotiflorin showed better chronic tolerance. Neither compound exhibited hepatotoxicity, and both compounds were non-sensitizers. The *T. pyriformis* toxicity value for beta-amyrin was 0.388, which is low. Nicotiflorin had a value of 0.285, slightly lower than that of beta-amyrin. Thus, both compounds had low toxicity in *T. pyriformis*.

The minnow toxicity value for beta-amyrin was −1.747, indicating low toxicity to minnows. Nicotiflorin’s value was significantly higher at 7.767, suggesting significant tolerance to minnows. Thus, nicotiflorin showed better tolerance to minnows.

Both compounds exhibited low acute toxicity, were not hepatotoxic, and did not cause skin sensitization. Nicotiflorin showed higher tolerance in chronic exposure scenarios and significantly lower minnow toxicity.

### 2.3. MDS Analysis

After a successful run, the XMGRACE tool was used to generate GROMACS trajectory files [16] (Turner, 2005). The developed 2D plots were analyzed for the radius of gyration (Rg), root mean square deviation (RMSD), root mean square fluctuation (RMSF), and hydrogen bond during a 100-ns simulation. The beta-sitosterol–protease complex showed higher fluctuation than other complexes.

**Figure 9:**
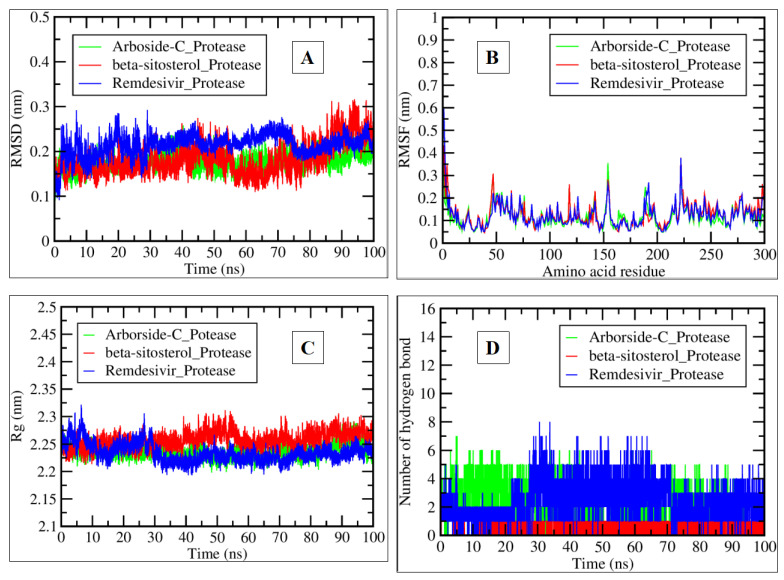
The plots generated by trajectory files. (**A**) RMSD plot of Arborside-C_Protease (green), Beta-Sitosterol_Protease (red), and Remdesivir_Protease (blue) complexes. (**B**) Root mean square fluctuation (RMSF) plot illustrating the volatility of each amino acid residue. (**C**) The radius of gyration (Rg) plot explaining the protease’s compactness, tightening in the presence of arborside-C, beta-sitosterol, and remdesivir. (**D**) Plot representing the interacting hydrogen bonds during a 100-ns simulation of selected complexes.

The deviation of the protease simulation in water and protease simulation in the presence of arborside-C, beta-sitosterol, and remdesivir was between 0.15 and 0.25 nm (Figure 9A). The Arborside-C_Protease complex showed a similar value (0.15) to that of the protease simulation in water and a lower RMSD value than the remdesivir–protease simulation value (more than 0.2 nm).

RMSF fluctuation values for complexes were between 0.1 and 0.35 nm (Figure 9B). All the chosen compounds had average RMSF values of around 0.1 nm at the 50–55, 120, 150–160, 188–190, 225–230, and 275–280 amino acid residue regions. A protein’s tertiary structure’s compactness and stability are indicated by its Rg values. When testing the stability of proteins in the presence of certain chemicals, this computation is essential, which was observed between 2.2 and 2.25 nm, as shown in the plot. At the same time, protease simulation in water and arborside-C and remdesivir presence in the system displayed numbers that were quite close (≥2.2 nm), while beta-sitosterol showed values near 2.25 nm (Figure 9C).

Hydrogen bonds are crucial in the interaction between ligands and proteins [17]. We generated a hydrogen bond plot. The Remdesivir_Protease complex formed eight hydrogen bonds compared to the Arborside-C_Protease complex, which included a maximum of seven bonds. In contrast, beta-sitosterol formed one hydrogen bond during the simulation period (Figure 9D).

**Figure 10:**
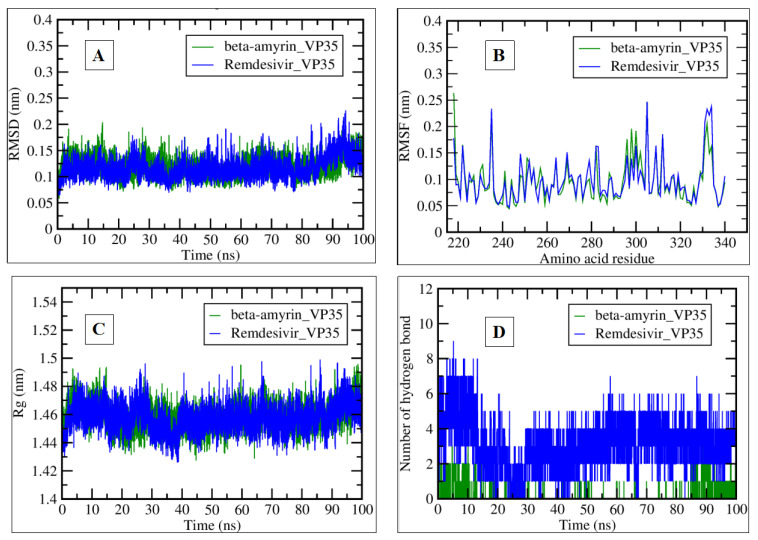
Plots generated by trajectory files. (**A**) RMSD plot of Beta-Amyrin_VP35 (green) and Remdesivir_VP35 (blue) complexes. (**B**) RMSF explaining each amino acid residue fluctuation. (**C**) Rg plot representing Ebola VP35 compactness, tightening in the presence of beta-amyrin and remdesivir. (**D**) Plot showing hydrogen bond interactions during a 100-ns simulation of selected complexes.

Both beta-amyrin_VP35 and remdesivir_VP35 exhibited fluctuations in RMSD over time (Figure 10A). The RMSD values remained below 0.15 nm throughout the 100-ns simulation. Both compounds maintained structural stability during the simulation.

Remdesivir_VP35 showed more significant fluctuations in RMSF than beta-amyrin_VP35, with an average value between 0.05 and 0.1 nm (Figure 10B). Remdesivir and beta-amyrin experienced flexibility or variability in specific amino acid residues and showed a similar pattern throughout the simulation. Further, both compounds exhibited identical fluctuations in the Rg over time. Rg values remained between 1.46 nm and 1.48 nm (Figure 10C). The overall compactness of the protein structures was maintained during the simulation. Remdesivir_VP35 consistently exceeded the formation of hydrogen bonds 1–9, while beta-amyrin_VP35 formed 1–2 hydrogen bonds (Figure 10D). Remdesivir formed more hydrogen bonds with the protein, potentially influencing its stability and interactions.

**Figure 11:**
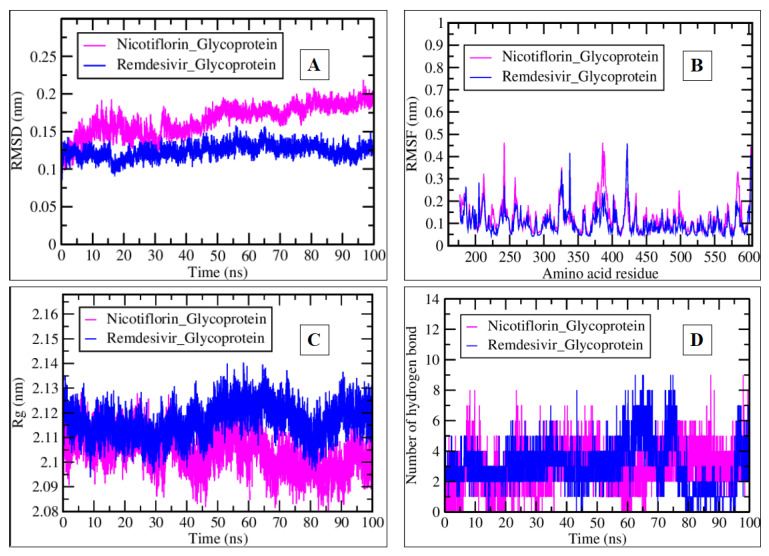
Plots generated by trajectory files. (**A**) RMSD graph of Nicotiflorin_Glycoprotein (pink) and Remdesivir_Glycoprotein (blue) complexes. (**B**) RMSF graph representing the fluctuation per amino acid residue. (**C**) Rg plot showing the Nipah glycoprotein tightening and compactness in the presence of nicotiflorin and remdesivir. (**D**) The number of hydrogen bond interactions during a 100-ns simulation of selected complexes.

We also analyzed similar MDS parameters to those of the MERS-CoV-2 protease receptor for Nicotiflorin_Glycoprotein and Remdesivir_Glycoprotein complexes of the Nipah glycoprotein. Both compounds exhibited similar behavior regarding RMSD over time. The RMSD values remained below 0.2 nm throughout the 100-ns simulation (Figure 11A). Both Nicotiflorin_Glycoprotein, shown at approximately 0.15 nm, and Remdesivir_Glycoprotein at 0.1 nm maintained structural stability during the simulation.

As shown in Figure 11B, the Nicotiflorin_Glycoprotein complex showed slightly higher fluctuations in RMSF across various amino acid residues compared to the Remdesivir_Glycoprotein. However, both complexes showed similar average values of 0.1 nm until the end of the simulation. Nicotiflorin_Glycoprotein exhibited greater flexibility or variability in specific protein structure regions (470–580 amino acids).

Both compounds exhibited similar fluctuations in Rg over time. Rg values remained between 2.10 nm and 2.13 nm (Figure 11C). The overall compactness of the protein structures was maintained during the simulation. Both Nicotiflorin_Glycoprotein and Remdesivir_Glycoprotein formed a significant number of hydrogen bonds. A total of 1–9 hydrogen bonds formed during the 100 ns simulation (Figure 11D). The fluctuations in hydrogen bonding patterns suggested dynamic interactions. Both compounds engaged in essential hydrogen bonding with the surrounding environment.

Overall, both compounds exhibited stable structures (low RMSD) and maintained compactness (consistent Rg). Nicotiflorin_Glycoprotein showed greater flexibility (higher RMSF) but formed a comparable number of hydrogen bonds. Remdesivir_Glycoprotein maintained stability and formed a similar number of hydrogen bonds. The differences in RMSF and hydrogen bonding may influence the binding affinities and interactions with the protein. In summary, Nicotiflorin_Glycoprotein and Remdesivir_Glycoprotein interacted differently with the protein, and their distinct features may impact their antiviral potential. Further studies are needed to understand their mechanisms and fully guide drug development.

Furthermore, we analyzed the time evolution of various properties of beta-amyrin’s and remdesivir’s interactions with chikungunya protease during MDS.

**Figure 12:**
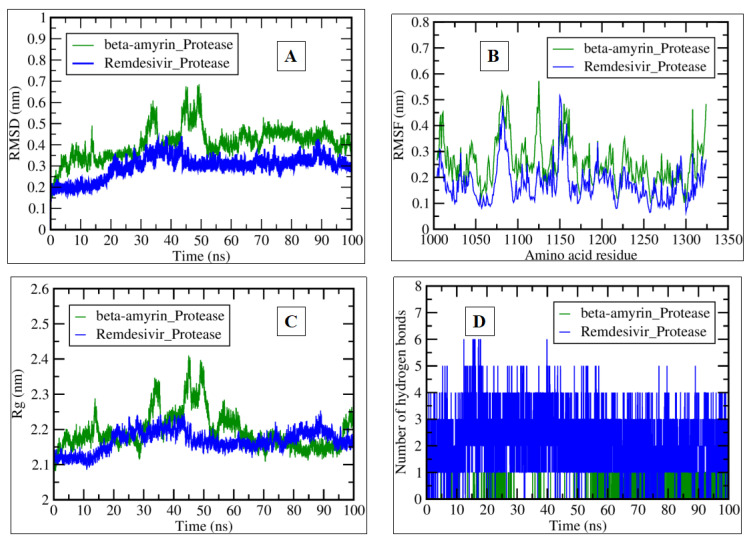
Plots generated by trajectory files. (**A**) RMSD plot of Beta-Amyrin_Protease (green) and Remdesivir_Protease (blue) complexes. (**B**) RMSF plot showing the per amino acid residue fluctuation. (**C**) Rg graph showing the chikungunya protease tightening and compactness in the presence of beta-amyrin and remdesivir. (**D**) Interacting hydrogen bonds during a 100-ns simulation of selected complexes.

The RMSD of Beta-Amyrin_Protease and Remdesivir_Protease relative to their initial structures over time (ns) were assessed. The RMSD of both complexes increased slowly over the course of the simulation, with remdesivir showing a slightly lower RMSD than beta-amyrin, with values ranging from 0.2 to 0.4 nm. This suggests that beta-amyrin undergoes slightly larger structural fluctuations than remdesivir during the simulation (Figure 12A). The RMSF of both molecules fluctuated around 0.1–0.2 nm throughout the simulation. This suggests that beta-amyrin and remdesivir have relatively stable backbone structures with minor fluctuations (Figure 12B). The Rg of Beta-Amyrin_Protease and Remdesivir_Protease over time (100 ns) was assessed. The Rg of both molecules fluctuated slightly over the course of the simulation, with remdesivir having a marginally higher average Rg compared to beta-amyrin at the end of the simulation, with a value between 2.1 and 2.2 nm. This suggests that remdesivir and beta-amyrin may have adopted a more extended conformation during the simulation (Figure 12C). Hydrogen bonds formed in the interaction between the protein and beta-amyrin and protease and remdesivir protease over time (ns). The number of hydrogen bonds between remdesivir and the protein fluctuated around 1–6 throughout the simulation, while the number of hydrogen bonds between beta-amyrin and the protein fluctuated around 2–3 (Figure 12D).

Overall, the results suggest that both beta-amyrin and remdesivir interact with the protein target. However, beta-amyrin appears to form a more stable interaction with the protein, likely due to a combination of factors such as smaller structural fluctuations and a higher number of hydrogen bonds.

MDS trajectory files were employed in the MMPBSA analysis process. Table 5 and Table 6 contain the results of the Poisson–Boltzmann complex energy and ligand–receptor energy component calculations, respectively. The total free binding energy for the given complexes is based on the provided table. Each component contributes to the overall binding energy and understanding these values sheds light on the stability and interactions within the complexes, as shown in Table 5.

Remdesivir showed −4799.56 (±26.97), −2462.65 (±0.74), −6959.81 (±1.38), and −7879.00 (±1.23) kcal/mol ΔGTotal against MERS-CoV-2 protease, Ebola VP35, Nipah glycoprotein, and chikungunya protease, respectively. The binding energy was predominantly influenced by electrostatic interactions (ΔEEL) and polar solvation energy (ΔEPB). The nonpolar contributions (ΔENPOLAR and ΔEDISPER) were relatively small. The gas phase energy (∆GGas) was negative, indicating favorable binding. Arborside-C, beta-sitosterol, and beta-amyrin with VP35 and nicotiflorin and beta-amyrin with chikungunya protease showed values of −4879.92 (±21.24), −4902.66 (±21.24), −2406.25 (±0.78), −12,777.97 (±1.46), and −7891.41 (±1.21) kcal/mol ΔGTotal. Like remdesivir, other compounds’ binding energies were mainly driven by electrostatic interactions and polar solvation. Nonpolar contributions played a minor role. The gas phase energy (∆GGas) was also negative. These complexes exhibited strong binding energies due to favorable electrostatic interactions and solvation effects. The gas phase energies indicated that these ligands were energetically stable when bound to their respective targets.

Table 5 summarizes the calculated data of Poisson–Boltzmann complex energy components ± standard error of the mean (SEM) of complexes with Remdesivir_MERS-CoV-2_Protease, Arborside-C_Protease, Beta-Sitosterol_Protease, Remdesivir_VP35 (Ebola), Beta-Amyrin_VP35 (Ebola), Remdesivir_Glycoprotein, (Nipah), Nicotiflorin_Glycoprotein (Nipah), Remdesivir_Protease (Chikungunya), and Beta-Amyrin_Protease (Chikungunya).

Here, ΔVdwaals = Van der Waals energy; ΔEPB = Polar contribution to the solvation energy; ΔEEL = Electrostatic molecular energy; ΔENPOLAR = Nonpolar contribution of repulsive solute–solvent interactions to the solvation energy; ΔG Gas = Total gas phase molecular energy; ΔEDISPER = Nonpolar contribution of attractive solute–solvent interactions to the solvation energy; ΔG Solv = Total solvation energy, and ΔG Total = Total binding energy.

The total binding energy (ΔGTotal) was negative for all complexes, indicating that the binding was favorable. However, the binding energy’s efficacy varied between the complexes.

The MMPBSA results showed that Remdesivir_MERS-CoV-2_Protease had a ΔGTotal of −22.63 (±0.74). Remdesivir showed a favorable binding energy, suggesting potential effectiveness against MERS-CoV-2 protease. Arborside-C_Protease showed a ΔGTotal of −16.30 (±1.16) and exhibited binding energy, indicating possible interactions with proteases. Beta-Sitosterol_Protease complex showed a ΔGTotal of −22.09 (±0.67). Beta-sitosterol’s binding energy also suggests interactions with proteases.

Remdesivir_VP35 (Ebola) complex showed a ΔGTotal of −26.23 (±0.12). Remdesivir’s binding energy implies a potential interaction with Ebola VP35. Beta-Amyrin_VP35 (Ebola) showed a slightly higher ΔGTotal of −28.10 (±0.10). This indicates that beta-amyrin also has binding energy, which indicates the potential for interaction with Ebola VP35. Remdesivir_Glycoprotein (Nipah) showed a ΔGTotal of −18.13 (±0.11). Remdesivir’s binding energy suggests interaction with Nipah glycoprotein. Nicotiflorin_Glycoprotein (Nipah) showed a ΔGTotal of −18.16 (±0.12). Nicotiflorin exhibited stronger interaction capabilities with the Nipah glycoprotein, similar to remdesivir. Remdesivir_Protease (Chikungunya) complex showed a ΔGTotal of −30.34 (±0.12); Beta-Amyrin_Protease (Chikungunya) also showed a very similar ΔGTotal of −29.14 (±0.09).

## 3. Discussion

Beta-amyrin showed binding energy, suggesting interactions with the chikungunya protease. It can be concluded that the presence of arborside-C and remdesivir in the target system stabilizes the protein, as evidenced by the lower RMSD and Rg values. In contrast, beta-sitosterol destabilizes the protein, as evidenced by the higher RMSD and Rg values. The hydrogen bond analysis also supports this conclusion. The Remdesivir_Protease complex formed eight hydrogen bonds, while the Arborside-C_Protease complex formed a maximum of seven bonds. In contrast, the Beta-Sitosterol_Protease complex formed only one hydrogen bond. These results suggest that arborside-C and remdesivir may be more effective protease inhibitors than beta-sitosterol. Further research is needed to confirm these findings.

More and more plant-based medications are used because of their therapeutic benefits and essential roles in achieving positive health outcomes [18]. Many plant compounds have been analyzed through different approaches to explore their interacting potential with viral proteases [5]. Numerous medicinal plants are rich in flavonoids and polyphenolic chemicals that have been shown to have anti-protease efficacy against multiple infections like hepatitis, flu, HIV, cytomegalovirus, herpes, smallpox, and Ebola [19,20,21]. Their antiviral activity could involve inhibiting reverse transcriptase enzyme proteases and DNA polymerase.

Compared to the native ligand, Mpro and other natural compounds studied, such as 2,2-di(3-indolyl)-3-indolone, terrequinone A, and betulinic acid, have demonstrated a greater affinity for binding. An anthraquinone from the observed list, alterporriol-Q, was discovered to be the most effective inhibitor of SARS-CoV-2 Mpro. Additionally, MD modeling experiments for the Mpro complex with alterporriol-Q indicate that alterporriol-Q does not significantly change the structure of Mpro [22,23,24].

In diverse *Nyctanthes arbortristis* tissues, steroids, flavonoids, terpenes, aliphatic chemicals, and alkaloids were found—the two primary types of chemicals made by this medicinal plant are glycosides and alkaloids. Several studies have focused on plant extracts from different parts of the plant for their therapeutic effects, such as anti-inflammatory, anti-allergic [25], antibacterial, antioxidant [26], anti-diabetic, anti-cancer, antiviral, and antimalarial properties [27]. The antiviral potential of arbortristoside A and C molecules against encephalomyocarditis virus and Semliki forest virus has been explored [28]. The studied bioactive molecules were observed to be more potent than the antiviral medication remdesivir. Arbrorside-C and beta-sitosterol have very good interaction energies, and these molecules significantly decreased protease-mediated infectivity. Recently, dietary bioactive compounds from three ayurvedic medicinal herbs, vasicoline (ΔG  =  −7.34 kcal/mol), solanocapsine (ΔG  =  −8.59 kcal/mol), and 18 β-glycyrrhetinic acid (ΔG  =  −8.86 kcal/mol) were reported to have significant binding interactions to Mpro enzyme compared to the native N3 inhibitor (ΔG  =  −5.41 kcal/mol) remdesivir. This supported our observed results, as the binding energies were much higher than those of the reported flavonoids. The binding of the molecules could be at the receptor binding domain sites at the His41 and Cys145. Quercetin and protease interactions were reported to be influenced by the presence of the 7-OH group and the acetylation of the sugar moiety. Moreover, the binding was based on their computer study of catechin gallate and quercetin 3-O-malonylglucoside, which are reported to inhibit the main protease by interacting with the active sites of the target protein (His41, Met49, Cys145, Met165, and Thr190) [29]. Researchers have recently explored the antiviral potential of beta-amyrin. This study has also shown that beta-amyrin interacted with viral protease, albeit with less potency than VP35, 3tkr chikungunya protease. Nicotiflorin interacts strongly with the Nipah G attachment glycoprotein. This suggests that nicotiflorin has medical uses, such as cardiac activity, which could be important in protecting the life of a heart patient who is infected with a virus [30,31].

Drug-likeness, molecular dynamics, and ADMET analysis of selected compounds represented important data more significantly than the remdesivir. The docking ADME results indicate the therapeutic significance of the studied antiviral molecules, which need further investigation.

## 4. Materials and Methods

### 4.1. Ligand Preparation

The 2D conformational information, including SMILES IDs, of the most significant 26 natural compounds of the plant *N. arbor-tristis* and control drug remdesivir was downloaded from the PubChem database [32] and utilized by converting it into 3D structural files for further molecular docking and simulation purposes. Furthermore, generated ligand files were submitted to the Discovery Studio Visualizer version 24.1.0.23298 for the energy minimization process using the CHARMm forcefield [33], which signifies the functional empirical energy to treat macromolecular systems as a model [34,35,36,37].

### 4.2. Viral Receptor Preparation

The target COVID-19 main protease (PDB ID: 6LU7) [38], Ebola virus VP35 (PDB ID: 4IBB) [39], and Nipah G attachment glycoprotein (PDB ID: 3D11) [40] and the structure of the chikungunya virus nsP2 protease (PDB ID: 3TRK) [41] were downloaded from the Protein Data Bank (PDB) database [42]. We removed the published structures’ HETATM and water molecules and applied the CHARMm forcefield [33]. The native 3D structural conformation of selected receptors was also examined, and amino acid residues of active sites involved in binding with pre-bounded ligands were discovered. These amino acid residues were then considered for docking studies. With the aid of Discovery Studio Visualizer version 24.1.0.23298, PDB files were rendered in 3D [34,35].

### 4.3. Molecular Docking Studies

To estimate the binding significance between the receptor structures of the different viruses and natural compounds, the MGL tool AutoDock 4.2, the Lamarckian genetic algorithm (LGA), and energy ∆G were calculated, as described by Morris [43,44]:

∆G binding = ∆Ggauss + ∆Grepulsion + ∆Ghbond + ∆Ghydrophobic + ∆Gtors

Here, the dispersion of two Gaussian functions in this instance has an attractive term of ∆G gauss. Repulsion: if the distance is less than a certain threshold, the square of the distance Ramp function for ∆Ghbond: also applied to interactions between metal ions ∆Ghydrophobic: ramp characteristic, ∆Gtors: represents the bond numbers that can be rotated.

The target molecules were dehydrated, and Kollman united, Gasteiger charges, and hydrogen were added to perform a docking with a point spacing of 0.375 by default in the grid box specification of 60 × 60 × 60° for X, Y, and Z with a grid center: for COVID-19 protease (PDB ID: 6LU7), −13.721, 16.337, and 69.721; for Ebola virus VP35 (PDB ID: 4IBB), 1.637, 21.039, and 1.327; for the crystal structure of the Nipah G attachment glycoprotein (PDB ID: 3D11), 35.436, 11.123, and 102.638; and for the structure of the chikungunya virus nsP2 protease (PDB ID: 3TRK), 12.76, 21.994, and 23.723, respectively. The parameters like the number of individuals in the population for Genetic Algorithm (150), the maximum number of energy evaluations (2,500,000), the maximum number of generations (27,000), the number of top individuals to survive to the next generation (1), the rate of gene mutation (0.02), the rate of crossover (0.8), the iterations of Solis and Wets local search (300), the consecutive successes before changing rho (0.1), the consecutive failures before changing rho (4), the probability of performing a local search on an individual (0.6), and the total 10 run were finally set for execution. The last obtained 3D conformations of the complexes were visualized in Discovery Studio Visualizer version 24.1.0.23298 [34,45].

### 4.4. Drug-Likeness and ADMET

The SwissADME and pkCSM online servers were used (http://www.swissadme.com, accessed on 1 March 2024), and drug-likeness and ADMET analyses of selected natural compounds in the computational mode were completed through the online resource provided by the Swiss Institute of Bioinformatics (SIB), Lausanne, Switzerland [45,46,47].

### 4.5. MDS

Natural compound–receptor complexes’ docking interactions were further assessed through MDS analysis; consequently, the MDS environment was configured to run simulations for these complexes for 100 ns using GROMACS [48].

The pdb2gmx module was used to create receptor topology files, and then CHARMM27 all-atom force fields were chosen. In the following step, the SwissParam server-generated topology files for the selected natural compounds and control drug remdesivir were used [49]. A water-filled triclinic box model of the unit cell was constructed for the solvation process. The network was stabilized after including Na^+^ and Cl^−^ ions, leading to a decrease in potential. After reaching harmony, the system (chosen complexes) was subjected to the NVT and NPT two-step arrays (constant particles, temperature, and pressure). Both ensembles’ pressure and temperature coupling can be controlled, allowing for more accurate simulations and excellent system stability [50]. The RMSD calculation tool integrated into the GROMACS, that is, the gmx rms package, was utilized to evaluate complex trajectory datasets [51], gmxrmsf for the calculation of RMSF, gmxgyrate for the Rg [51], and gmxhbond to determine how fast hydrogen bonds form throughout the exchange. The Xmgrace tool was used to make 2D plots [16].

Furthermore, the Molecular Mechanics–Poisson–Boltzmann Surface Area (MM-PBSA) [52] method-based free energy calculation was executed after utilizing the MMPBSA.py Python script developed by Miller et al. [53]. The free energy calculation was finally run by the gmx_MMPBSA program [54].

## 5. Conclusions

Effective antiviral therapies are urgently needed for the management of viral diseases due to viruses’ high transmission rates, mutagenicity, and genomic reorganization. The major Mpro enzymes, helicases, RNA-dependent RNA polymerase, and glycoproteins are examples of viral molecules that can be targeted with antiviral drugs to stop the replication and spread of viral infections. Viruses are extremely mutagenic, although any genetic change to the highly conserved protease enzyme frequently has adverse effects on them. Thus, using protease enzyme inhibitors may lower the probability of mutation-mediated medication resistance and offer an efficient antiviral defense. Several currently available antiviral medications and dietary bioactive agents are used against viruses. We used computational tools and methods to determine how arborside-C, beta-sitosterol, and beta-amyrin from *Nyctanthes arbortristis* bound to targets on Ebola virus VP35, SARS-CoV-2 protease, Nipah virus glycoprotein, and chikungunya virus. According to ADMET data, the characteristics of arborside-C and the reference medication remdesivir are similar. For the complexes of phytochemicals with targets, additional MDSs produced encouraging outcomes. RMSD, as observed in nm, signified the stability of the complexes. Based on our data, the phytochemicals of *Nyctanthes arbor tristis* could be recognized as excellent broad-spectrum natural antiviral chemicals. Our findings suggest that these substances could be used as drugs to treat viral diseases of various origins. These chemicals may be candidates for treatment regimens following in vitro and in vivo clinical trials.

## Figures and Tables

**Table 1:** Molecular interaction analysis details the screened tops of two natural compounds and the main protease of a SARS-CoV-2 crystal structure (PDB: 6LU7). In the “number of hydrogen bonds” column, UNK1 = ligand molecules selected for the interaction.

Compound Name	Residues Involved in Hydrophobic Interactions	Inhibition Constant (Ki)	Hydrogen Bond Length (Angstrom)	No. of Hydrogen Bonds	Binding Energy (kcal/mol)	Compound Name
Remdesivir	Gly138, Ser144, His164, His172	166.35 μM	2.81	:UNK1:H70 - A:SER139:OG	−6.18	Alkyl/Pi-Alkyl = Leu141, Cys145, His163, Met165 attractive charge Glu166
			2.28	:UNK1:H60 - A:ASN142:OD1		
			3.15	:UNK1:C16 - A:PHE140:O		
			2.84	:UNK1:C16 - A:GLU166:OE2		
			2.81	:UNK1:H70 - A:SER139:OG		
Arborside-C	Thr25, Leu27, His41, Met49, Ser144, Cys145, His164, Met165	456.39 nM	2.24	A:THR26:HN - :UNK1:O30	−8.65	Pi-Alkyl = His172
			1.98	A:GLY143:HN - :UNK1:O19		
			2.16	A:HIS163:HE2 - :UNK1:O35		
			2.99	A:GLN189:HE21 - :UNK1:O9		
			2.06	:UNK1:H61 - A:THR26:O		
			3.4	:UNK1:C18 - A:LEU141:O		
			2.86	:UNK1:C28 - A:THR26:O		
			3.14	:UNK1:C36 - A:PHE140:O		
			3.47	:UNK1:C36 - A:GLU166:OE2		
Beta-sitosterol	Thr25, Met49, Asn142, Gly143, Ser144, His164, Glu166, Pro168, Leu167, Gln189, Arg188, Thr190, Gln192	210.29 nM	1.88	:UNK1:H67 - A:THR26:O	−9.11	Alkyl/Pi-Alkyl Leu27, His41, Cys145, Met165

**Table 2:** Molecular interaction analysis details the screened top natural compound with control drug remdesivir and Ebola virus VP35 bound to a small molecule (PDB: 4IBB). In the “number of hydrogen bonds” column, UNK = ligand molecules selected for the interaction.

Compound Name	Residues Involved in Hydrophobic Interactions	Inhibition Constant (Ki)	Hydrogen Bond Length (Angstrom)	No. of Hydrogen Bonds	Binding Energy (kcal/mol)	Residues Involved in Other Interactions
Remdesivir	Val221, Val245, Gln244, Val294, Pro293, Asp302, Phe328	1.21 mM	2.33	A:GLN241:HE22 - A:UNK0:N23	−3.98	Pi-Sigma = Lys248 Alkyl/Pi-Alkyl = Ile295, Ile297
			2.09	A:UNK0:H60 - A:HIS296:O		
			3.11	A:HIS296:HN - A:UNK0		
Beta-amyrin	Ala221, Gln241, Phe328, Asp302, Ile303, Ile297	3.08 μM	2.05	A:ARG225:HH11 - :UNK1:O24	−7.52	Alkyl = Cys247, Lys248, Ile295, Pro304

**Table 3:** Molecular interaction analysis details the screened top natural compound with the control drug remdesivir and the crystal structure of the Nipah G attachment glycoprotein (PDB ID: 3D11). In the “number of hydrogen bonds” column, UNK = ligand molecules selected for the interaction.

Compound Name	Residues Involved in Hydrophobic Interactions	Inhibition Constant (Ki)	Hydrogen Bond Length (Angstrom)	No. of Hydrogen Bonds	Binding Energy (kcal/mol)	Residues Involved in Other Interactions
Remdesivir	Pro353, Gly352, Cys282, Tyr351, Phe458, Trp504, Leu305, Arg242, Asp219, Pro220, Val507, Thr218, Ser241, Leu234	25.29 μM	2.65	A:ARG236:HH22 - A:UNK0:O9	−6.27	Pi-Alkyl = Tyr280, Pro441 Pi-Sigma = His281, Pi-Cation = Lys560, Pi-Anion = Asp302 Attractive charge = Asp302
			2.28	A:UNK0:H71 - A:GLU579:OE1		
			2.2	A:UNK0:H72 - A:GLU579:OE2		
			3.39	A:UNK0:C18 - A:GLN559:OE1		
			2.26	A:LYS560:HZ2 - A:UNK0		
Nicotiflorin	Asn586, Asp302, Gly301, Tyr280, Leu305, Thr218, Pro220	20.63 μM	1.87	A:ASP219:HN - :LIG1:O36	−6.39	Alkyl/Pi-Alkyl = Ile588, Tyr581
			2.85	A:ARG236:HH21 - :LIG1:O19		
			1.93	A:CYS240:HN - :LIG1:O41		
			2.55	A:ARG242:HH22 - :LIG1:O28		
			2.69	:LIG1:H66 - A:GLU579:OE2		
			2.66	:LIG1:H70 - A:SER239:OG		
			2.7	:LIG1:H69 - A:GLN559:OE1		
			2.9	A:SER241:CB - :LIG1:O15		
			3.4	A:SER241:CB - :LIG1:O8		

**Table 4:** Molecular interaction analysis detailing the screened top natural compound with the control drug remdesivir and the structure of the chikungunya virus nsP2 protease (PDB ID: 3TRK). In the “number of hydrogen bonds” column, UNK = ligand molecules selected for the interaction.

Compound Name	Residues Involved in Hydrophobic Interactions	Inhibition Constant (Ki)	Hydrogen Bond Length (Angstrom)	No. of hydrogen bonds	Binding Energy (kcal/mol)	Residues Involved in Other Interactions
Remdesivir	His1236, Ala1237, Lys1045, Tyr1047, Tyr1079, Leu1205, Trp1084, Pro1049, Lys1091, Glu1050, Arg1271, Thr1268, Leu1243, Glu1204	95.82 μM	2.62	A:GLN1241:HE21 - A:UNK0:O15	−5.48	Alkyl/Pi-alkyl = Lys1239, Ala1046, Val1051
			2.1	A:UNK0:H70 - A:ASP1235:O		
			3.05	A:SER1048:CB - A:UNK0:O36		
Beta-amyrin	Val1051, Gln1241, Leu1205, Gly1206, Glu1204, Leu1243, Leu1203	551.06 nM	2.11	:UNK1:H63 - A:SER1048:OG	−8.54	Alkyl/Pi-alkyl = Tyr1079, Trp1084, Al1046, Tyr1047, Ala1040, Lys1239, Lys1045

**Table 5:** Summary of the calculated data of Poisson–Boltzmann complex energy components ± standard error of the mean (SEM) of complexes with Remdesivir_MERS-CoV-2_Protease, Arbor-side-C_Protease, Beta-Sitosterol_Protease, Remdesivir_VP35 (Ebola), Beta-Amyrin_VP35 (Ebola), Remdesivir_Glycoprotein, (Nipah), Nicotiflorin_Glycoprotein (Nipah), Remdesivir_Protease (Chikungunya), and Beta-Amyrin_Protease (Chikungunya).

Complex Free Energy Calculation Components								
Complex	ΔVdwaals	ΔEEL	ΔEPB	ΔENPOLAR	ΔEDISPER	∆GGas	∆GSolv	∆GTotal
**Remdesivir_MERS−CoV−2_Protease**	−2176.97 (±17.11)	−18,897.97 (±39.08)	−3003.96 (±33.10)	74.28 (±0.37)	0.00 (±0.0)	−1869.88 (±59.08)	−2929.68 (±32.79)	−4799.56 (±26.97)
**Arborside−C_Protease**	−2149.23 (±5.80)	−18,756.90 (±54.05)	−2945.80 (±29.29)	73.80 (±0.34)	0.00 (±0.0)	−2007.92 (±43.40)	−2872.00 (±29.07)	−4879.92 (±21.24)
**Beta−Sitosterol_Protease**	−2111.24 (±18.17)	−18,839.26 (±75.99)	−2927.12 (±34.55)	73.58 (±0.51)	0.00 (±0.00)	−2049.11 (±72.59)	−2853.54 (±34.05)	−4902.66 (±41.16)
**Remdesivir_VP35** **(Ebola)**	−847.72 (±0.31)	−6939.06 (±1.89)	−1966.78 (±1.71)	36.88 (±0.01)	0.00 (±0.00)	−532.75 (±1.89)	−1929.90 (±1.71)	−2462.65 (±0.74)
**Beta−Amyrin_VP35** **(Ebola)**	−820.95 (±0.33)	−6792.64 (±2.04)	−1874.47 (±1.85)	35.83 (±0.01)	0.00 (±0.00)	−567.62 (±2.15)	−18.38.63 (±1.85)	−2406.25 (±0.78)
**Remdesivir_Glycoprotein** **(Nipah)**	−3049.62 (±0.60)	−26,201.89 (±2.98)	−4002.48 (±2.22)	94.81 (±0.02)	0.00 (±0.00)	−3052.14 (±2.79)	−3907.67 (±2.21)	−6959.81 (±1.38)
**Nicotiflorin_Glycoprotein** **(Nipah)**	−3527.83 (±0.69)	−30,610.79 (±2.23)	−3952.60 (±1.65)	90.44 (±0.03)	0.00 (±0.00)	−8915.81 (±2.31)	−3862.16 (±1.64)	−12,777.97 (±1.46)
**Remdesivir_Protease** **(Chikungunya)**	−2147.94 (±0.52)	−16,269.22 (±2.11)	−4631.77 (±1.59)	85.68 (±0.03)	0.00 (±0.00)	−3332.91 (±2.08)	−4546.08 (±2.08)	−7879.00 (±1.23)
**Beta−Amyrin_Protease** **(Chikungunya)**	−2108.04 (±0.54)	−15,915.65 (±2.21)	−4760.74 (±1.75)	87.71 (±0.03)	0.00 (±0.00)	−3218.38 (±2.20)	−4673.03 (±1.74)	−7891.41 (±1.21)

**Table 6:** Summary of MMPBSA-based free energy calculation components ± SEM of ligand–receptor with Remdesivir_MERS-CoV-2_Protease, Arborside-C_Protease, Beta-Sitosterol_Protease, Remdesivir_VP35 (Ebola), Beta-Amyrin_VP35 (Ebola), Remdesivir_Glycoprotein, (Nipah), Nicotiflorin_Glycoprotein (Nipah), Remdesivir_Protease (chikungunya), and Beta-Amyrin_Protease (chikungunya).

Ligand–Receptor Free Energy Calculation Components								
Complex	ΔVdwaals	ΔEEL	ΔEPB	ΔENPOLAR	ΔEDISPER	∆GGas	∆GSolv	∆GTotal
**Remdesivir_MERS-CoV-2_Protease**	−43.38 (±0.54)	−10.79 (±3.74)	36.02 (±3.17)	−4.47 (±0.04)	0.00 (±0.00)	−54.18 (±3.51)	31.55 (±3.14)	−22.63 (±0.74)
**Arborside-C_Protease**	−34.44 (±0.93)	−11.90 (±2.06)	34.33 (±2.08)	−4.30 (±0.14)	0.00 (±0.00)	−46.34 (±2.50)	30.04 (±1.98)	−16.30 (±1.16)
**Beta-Sitosterol_Protease**	−31.78 (±0.64)	−5.10 (±0.41)	18.60 (±0.19)	−3.80 (±0.07)	0.00 (±0.00)	−36.89 (±0.64)	14.80 (±0.19)	−22.09 (±0.67)
**Remdesivir_VP35** **(Ebola)**	−34.01 (±0.08)	−17.07 (±0.14)	28.55 (±0.12)	−3.69 (±0.01)	0.00 (±0.00)	−51.08 (±0.16)	24.85 (±0.12)	−26.23 (±0.12)
**Beta-Amyrin_VP35** **(Ebola)**	−34.93 (±0.07)	−5.54 (±0.15)	16.04 (±0.08)	−3.66 (±0.00)	0.00 (±0.00)	−40.47 (±0.16)	12.38 (±0.18)	−28.10 (±0.10)
**Remdesivir_Glycoprotein** **(Nipah)**	−26.61 (±0.10)	−21.42 (±0.47)	32.99 (±0.39)	−3.09 (±0.01)	0.00 (±0.00)	−48.03 (±0.45)	29.90 (±0.38)	−18.13 (±0.11)
**Nicotiflorin_Glycoprotein** **(Nipah)**	−35.37 (±0.06)	−28.22 (±0.24)	48.94 (±0.23)	−3.96 (±0.00)	0.00 (±0.00)	−63.59 (±0.24)	44.98 (±0.23)	−18.16 (±0.12)
**Remdesivir_Protease** **(Chikungunya)**	−52.91 (±0.09)	43.85 (±0.21)	71.67 (±0.16)	−5.25 (±0.01)	0.00 (±0.00)	−96.76 (±0.22)	66.42 (±0.16)	−30.34 (±0.12)
**Beta-Amyrin_Protease** **(Chikungunya)**	−39.15 (±0.10)	−6.18 (±0.06)	20.25 (±0.06)	−4.06 (±0.01)	0.00 (±0.00)	−45.33 (±0.11)	16.19 (±0.06)	−29.14 (±0.09)

## Data Availability

Data are contained within the paper and the Supplementary Materials.

## Supplementary Materials

The following supporting information can be downloaded at https://www.mdpi.com/article/10.3390/ph17050581/s1, Table S1: Molecular interaction analysis details between screened natural compounds and Crystal structure of SARS-CoV2 main protease (PDB:6LU7)). In the column hydrogen bond details where UNK1 = selected natural compounds title; Table S2: ADME prediction from SwissADME (GI = Gastro intestinal, BBB = Blood Brain Barrier, Pgp = P glycoprotein, CYP = Cytochrome, log Kp = skin permeation); Table S3: Drug-likeness prediction from SwissADME server (MW = Molecular Weight, TPSA = total polar surface area, Consensus Log P = average of all predicted Log Po/w; Table S4: toxicity prediction, Data obtained from pkCSM server.
