# Supplementary material for: Investigating the Antiviral Properties of Nyctanthes arbor-tristis Linn against the Ebola, SARS-CoV-2, Nipah, and Chikungunya Viruses: A Computational Simulation Study
Source: Pharmaceuticals (Basel). 2024 Apr 30;17(5):581. doi: 10.3390/ph17050581 (PMC11124395; doi:10.3390/ph17050581)
Supplement: Supplementary file 1 [file pharmaceuticals-17-00581-s001.zip › pharmaceuticals-2976707-supplementary.pdf]

Table S1: **Molecular interaction analysis details between screened natural compounds and Crystal structure of SARS-CoV2 main protease (PDB:6LU7)). In the column hydrogen bond details where UNK1=selected natural compounds**

S.No.	Compound Name	Binding energy	Hydrogen bond name	Hydrogen bond Length	Inhibition constant	Residues involved in hydrophobic interaction
1.	alpha-Pinene	-5.33	Not formed	Not formed	122.91 uM	His41<br>Met49<br>Tyr54<br>His164<br>Met165<br>Asp187<br>Arg188<br>Gln189
2.	Alpha-xylopyranoside	-5.95	A:SER144:HG - :UNK1:O21	3.00218	43.78 uM	Thr25<br>Thr26<br>Leu27<br>His41<br>Met49<br>Phe140<br>Leu141<br>Asn142<br>Gly143<br>Ser144<br>Cys145<br>His163<br>His164
			A:CYS145:HN - :UNK1:O17	2.97309		
			A:CYS145:SG - :UNK1:O6	3.23842		
			A:HIS163:HE2 - :UNK1:O21	1.96749		
			:UNK1:H40 - A:ASN142:OD1	2.08201		
			:UNK1:H41 - A:LEU141:O	2.95556		
			:UNK1:H35 - A:THR26:O	2.39324		
			:UNK1:H37 - A:THR26:O	2.5729		

			:UNK1:H36 - A:THR26:O	2.24898		
			:UNK1:C1 - A:LEU141:O	2.72466		
			:UNK1:C1 - A:SER144:OG	3.02486		
3.	Apigenin	-7.29	A:GLY143:HN - :UNK1:O18	2.08523	4.54 uM	His41<br>Met49<br>Tyr54<br>Leu141<br>Asn142<br>Gly143<br>Ser144<br>Cys145<br>His163<br>His164<br>Met165<br>Glu166<br>Asp187<br>Arg188<br>Gln189
			A:GLU166:HN - :UNK1:O10	1.96446		
			:UNK1:H30 - A:ASP187:O	1.82659		
			:UNK1:H28 - A:LEU141:O	1.98939		
			A:CYS145:SG - :UNK1	4.06905		
4.	Arborside-B	-7.96	A:SER144:HN - :UNK1:O29	2.08674	1.47 uM	His41<br>Leu141<br>Asn142<br>Gly143<br>Ser144<br>Cys145<br>His163<br>His164<br>Met165<br>Glu166
			A:CYS145:HN - :UNK1:O29	2.56942		
			:UNK1:H59 - A:ASN142:OD1	2.82963		
			:UNK1:H60 - A:ASN142:OD1	1.88472		
			A:GLY143:HN - :UNK1	2.91948		

			A:CYS145:SG - :UNK1	4.09983		Gln189
5.	Arborside-C	−8.65	A:THR26:HN - :UNK1:O30	2.24178	456.39 nM	Thr25<br>Thr26<br>Leu27<br>His41<br>Met49<br>Phe140<br>Leu141<br>Asn142<br>Gly143<br>Ser144<br>Cys145<br>His163<br>His164<br>Met165<br>Glu166<br>His172<br>Gln189
			A:GLY143:HN - :UNK1:O19	1.98872		
			A:HIS163:HE2 - :UNK1:O35	2.16799		
			A:GLN189:HE21 - :UNK1:O9	2.99984		
			:UNK1:H61 - A:THR26:O	2.0638		
			:UNK1:C18 - A:LEU141:O	3.40553		
			:UNK1:C28 - A:THR26:O	2.86457		
			:UNK1:C36 - A:PHE140:O	3.14551		
			:UNK1:C36 - A:GLU166:OE2	3.47377		
6.	Arbortristoside-B	−7.68	A:GLY143:HN - :UNK1:O40	2.79446	2.35 uM	Thr25<br>Thr26<br>Leu27<br>His41<br>Met49<br>Phe140<br>Leu141<br>Asn142<br>Gly143<br>Ser144
			A:CYS145:HN - :UNK1:O40	2.29093		
			A:CYS145:HN - :UNK1:O41	2.97845		
			A:HIS163:HE2 - :UNK1:O4	2.37026		
			:UNK1:H71 - A:THR26:O	2.08247		

			:UNK1:H73 - A:CYS145:SG	2.3045		Cys145<br>His163<br>His164<br>Met165<br>Glu166<br>Pro168<br>His172<br>Arg188<br>Gln189<br>Thr190<br>Ala191<br>Gln192
			:UNK1:H54 - A:GLN189:OE1	2.09123		
			:UNK1:H55 - A:ASN142:OD1	1.96576		
			:UNK1:H61 - A:THR190:O	2.0507		
7.	Arbortristoside-C	-7.50	A:HIS41:HE2 - :UNK1:O9	2.01607	3.19 uM	Thr24<br>Thr25<br>Thr26<br>His41<br>Asn142<br>Gly143<br>Cys145<br>His163<br>His164<br>Met165<br>Glu166<br>Arg188<br>Gln189<br>Thr190<br>Gln192
			A:CYS145:SG - :UNK1:O9	2.76755		
			A:GLU166:HN - :UNK1:O22	2.0587		
			:UNK1:H65 - A:GLN189:OE1	2.06957		
			:UNK1:H66 - A:ASN142:OD1	2.13344		
			:UNK1:H54 - A:THR24:O	2.14432		
			:UNK1:H55 - A:HIS164:O	1.80929		
			:UNK1:C39 - A:ARG188:O	2.88695		
			A:THR26:HN - :UNK1	2.86371		

8.	arbortristoside-D	−5.86	A:HIS163:HE2 - :UNK1:O38	2.08926	50.37 uM	Thr25<br>Thr26<br>Leu27<br>Met49<br>Phe140<br>Leu141<br>Asn142<br>Gly143<br>Ser144<br>Cys145<br>His163<br>Met165<br>Glu166<br>Leu167<br>Pro168<br>His172<br>Gln189
			A:HIS163:HE2 - :UNK1:O39	2.21217		
			:UNK1:H71 - A:LEU141:O	2.36987		
			:UNK1:H71 - A:SER144:OG	2.0504		
			:UNK1:H72 - A:CYS145:SG	2.51634		
			:UNK1:H59 - A:THR26:O	2.00564		
			:UNK1:H60 - A:THR26:O	1.76109		
			:UNK1:C6 - A:GLU166:O	3.14568		
			:UNK1:C31 - A:ASN142:OD1	3.1776		
			:UNK1:C37 - A:PHE140:O	3.09174		
			:UNK1:C37 - A:GLU166:OE2	2.98373		
9.	benzoic_acid	−3.73	A:TYR54:HH - :UNK1:O8	2.2045	1.85 mM	His41<br>Cys44<br>Met49<br>Leu50<br>Pro52<br>Tyr54<br>His164<br>Met165<br>Asp187
			:UNK1:H15 - A:MET49:O	1.94985		

						Arg188<br>Gln189
10.	beta-Sitosterol	-9.11 kcal/mol	:UNK1:H67 - A:THR26:O	1.88315	210.29 nM	Thr26<br>His41<br>Met49<br>Gly143<br>Ser144<br>Cys145<br>His164<br>Met165<br>Glu166<br>Arg188<br>Gln189<br>Thr190<br>Gln192
11.	hentriacontane	-4.41	Not formed	Not formed	589.57 uM	Thr24<br>Thr25<br>Thr26<br>Leu27<br>His41<br>Asn119<br>Phe140<br>Leu141<br>Asn142<br>Gly143<br>Ser144<br>Cys145<br>His163<br>His164<br>Met165<br>Glu166

12.	Kaemferol	-7.01	A:HIS163:HE2 - :UNK1:O18	1.88967	7.32 uM	His41 Met49 Phe140 Leu141 Gly143 Ser144 Cys145 His163 His164 Met165 Glu166 Arg188 Gln189 Thr190 Gln192
			A:GLU166:HN - :UNK1:O17	2.19162		
			:UNK1:H31 - A:ARG188:O	1.93641		
			:UNK1:H30 - A:GLN189:OE1	1.83423		
			:UNK1:H28 - A:LEU141:O	2.46306		
			A:CYS145:SG - :UNK1	4.00364		
13.	lupeol	-5.97	A:THR26:HN - :UNK1:O29	2.6656	42.41 uM	Thr24 Thr25 Thr26 Met49 Leu141 Asn142 Gly143 Ser144 Cys145 His163 His164 Met165 Glu166
			:UNK1:H75 - A:THR24:O	2.11616		
			A:THR25:CA - :UNK1:O29	2.78459		
14.	Mannitol	-4.13	A:GLY143:HN - :UNK1:O12	2.29082	946.30 uM	Leu141 Asn142

			A:CYS145:HN - :UNK1:O12	2.79644		Gly143 Ser144 Cys145 His163 Glu166 Gln189
			:UNK1:H26 - A:LEU141:O	1.88062		
			:UNK1:H26 - A:SER144:OG	2.95403		
			:UNK1:H25 - A:LEU141:O	2.03886		
			:UNK1:H21 - A:GLN189:OE1	2.10034		
			:UNK1:H22 - A:GLN189:OE1	1.92968		
			:UNK1:H24 - A:ASN142:OD1	2.18809		
15.	methyl_salicylate	-4.28	A:GLY143:HN - :UNK1:O11	3.09594	733.45 uM	Thr26 Leu27 His41 Leu141 Asn142 Gly143 Ser144 Cys145 His163 Met165 Glu166
			A:CYS145:HN - :UNK1:O2	2.29403		
			A:CYS145:HN - :UNK1:O11	2.45436		
			:UNK1:H19 - A:LEU141:O	1.95009		
			:UNK1:H19 - A:SER144:OG	1.85687		
			A:CYS145:SG - :UNK1	3.61396		
16.	Nyctanthic_acid	-7.09	A:THR26:HN - :UNK1:O15	1.84215	6.31 uM	Thr24 Thr25 Thr26 His41 Met49
			:UNK1:H51 - A:THR24:O	2.03147		

						Phe140<br>Leu141<br>Asn142<br>Gly143<br>Ser144<br>Cys145<br>His163<br>His164<br>Met165<br>Glu166
17.	Nyctanthoside	-5.65	A:HIS163:HE2 - :UNK1:O15	1.86791	72.48 uM	His41<br>Phe140<br>Leu141<br>Asn142<br>Ser144<br>His163<br>His164<br>Met165<br>Glu166<br>His172<br>Arg188<br>Gln189
			:UNK1:H52 - A:HIS164:O	1.97887		
			:UNK1:H54 - A:GLU166:O	2.14452		
			:UNK1:H55 - A:GLU166:O	2.22595		
			:UNK1:H42 - A:LEU141:O	2.2175		
			:UNK1:H43 - A:PHE140:O	2.6644		
			:UNK1:H43 - A:GLU166:OE2	1.72767		
			:UNK1:C23 - A:GLN189:OE1	3.28385		
			:UNK1:C1 - A:GLU166:OE1	3.38305		
18.	p-cymene	-4.72	NA	NA	349.63 uM	His41<br>Cys44<br>Met49

						Pro52<br>Tyr54<br>His164<br>Met165<br>Asp187<br>Arg188<br>Gln189
19.	Quercetin	−8.43	:UNK1:H32 - A:THR190:O	2.13572	664.97 nM	His41<br>Tyr54<br>Cys145<br>His164<br>Met165<br>Glu166<br>Leu167<br>Pro168<br>Asp187<br>Arg188<br>Gln189<br>Thr190<br>Ala191<br>Gln192
			:UNK1:H31 - A:THR190:O	2.32119		
			:UNK1:H30 - A:GLU166:O	2.061		
			:UNK1:H29 - A:HIS164:O	3.02736		
			:UNK1:H28 - A:ASP187:O	2.49028		

Table S2. ADME prediction from SwissADME (GI=Gastro intestinal, BBB=Blood Brain Barrier, Pgp=P glycoprotein, CYP=Cytochrome, log Kp= skin permeation)									
**Compounds**	**GI absorption**	**BBB permeant**	**Pgp substrate**	**CYP1A2 inhibitor**	**CYP2C19 inhibitor**	**CYP2C9 inhibitor**	**CYP2D6 inhibitor**	**CYP3A4 inhibitor**	**log Kp (cm/s)**
									negative the log K_p the less skin permeant the molecule
Remdesivir	Low	No	Yes	No	No	No	No	Yes	-8.62
Arborside-C	Low	No	Yes	No	No	No	No	No	-9.51
Beta-Sitosterol	Low	No	No	No	No	No	No	No	-2.2
Beta-amyrin	Low	No	No	No	No	No	No	No	-2.41
Nicotiflorin	Low	No	Yes	No	No	No	No	No	-9.91

**Table S3. Drug-likeness prediction from SwissADME server (MW=Molecular Weight, TPSA= total polar surface area, Consensus Log P= average of all predicted Log Po/w**													
**Compounds**	**MW (g/mol)**	**Rotatable bonds**	**H-bond acceptors**	**H-bond donors**	**TPSA (Å²)**	**Consensus Log P**	**Lipinski violations**	**Ghose violations**	**Veber violations**	**Egan violations**	**Muegge violations**	**Bioavailability Score**	**Synthetic Accessibility**
	MW between 150 and 500 g/mol	no more than 9 rotatable bonds			TPSA between 20 and 130 Å^2	not higher than 6						not less than 0.25	normalized between 1 (easy synthesis) and 10 (very difficult synthesis)
Remdesivir	602.58	14	12	4	213.36	1.54	2	3	2	1	3	0.17	6.43
Arborside-C	510.49	8	12	5	181.44	-0.31	2	2	1	1	2	0.11	6.14
Beta-Sitosterol	414.71	6	1	1	20.23	7.19	1	3	0	1	2	0.55	6.3
Beta-amyrin	426.72	0	1	1	20.23	7.18	1	3	0	1	2	0.55	6.04
Nicotiflorin	594.52	6	15	9	249.20	-0.73	3	4	1	1	3	0.17	6.48

Table S4: toxicity prediction. Data obtained from pkCSM server

	AMES toxicity	Max. tolerated dose (Human)	hERG I inhibitor	hERG II inhibitor	Oral Rat Acute Toxicity (LD50)	Oral Rat Chronic Toxicity (LOAEL)	Hepatotoxicity	Skin sensitisation	T. pyriformis toxicity	Minnow toxicity
**Standard cut-off**	**Positive mutagenic**	**<= 0.477 log(mg/kg/day)**				**lowest**			**> -0.5 log ug/L**	**Log LC50< -0.3**
**Compounds**										
Remdesivir	No	0.323	No	Yes	2.329	2.477	No	No	0.285	1.57
Arborside-C	No	-0.397	No	Yes	2.701	4.723	No	No	0.285	3.731
Beta-Sitosterol	Yes	0.559	No	No	2.482	-0.515	No	No	0.285	4.614
Beta-amyrin	No	-0.312	No	Yes	2.296	0.922	No	No	0.388	-1.747
Nicotiflorin	No	0.221	No	Yes	2.518	5.488	No	No	0.285	7.767
